# 5-Benzene­sulfonamido-2-chloro­benzoic acid

**DOI:** 10.1107/S1600536809009787

**Published:** 2009-03-25

**Authors:** Muhammad Nadeem Arshad, M. Nawaz Tahir, Islam Ullah Khan, Muhammad Shafiq, Hafiz Muhammad Adeel Sharif

**Affiliations:** a Department of Chemistry, Government College University, Lahore, Pakistan; b Department of Physics, University of Sargodha, Sargodha, Pakistan

## Abstract

In the title compound, C_13_H_10_ClNO_4_S, the dihedral angle between the aromatic ring planes is 87.07 (6)° and an intra­molecular C—H⋯O inter­action occurs. In the crystal, inversion dimers linked by two O—H⋯O hydrogen bonds arise from the carboxyl groups. N—H⋯O hydrogen bonds link the dimers into chains and short C—Cl⋯π and S—O⋯π contacts are also seen.

## Related literature

For related structures: see: Arshad *et al.* (2008 ▶); Arshad, Khan *et al.* (2009 ▶); Arshad, Tahir *et al.* (2009 ▶). For chemical background, see: Bouchain *et al.* (2003 ▶). For graph-set theory, see: Bernstein *et al.* (1995 ▶).
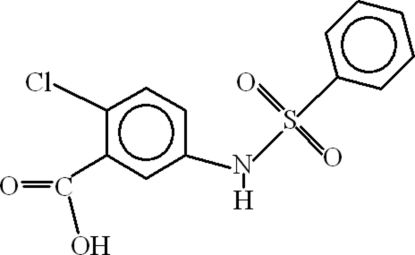


## Experimental

### 

#### Crystal data

C_13_H_10_ClNO_4_S
*M* *_r_* = 311.73
Monoclinic, 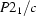

*a* = 11.7139 (4) Å
*b* = 5.3957 (2) Å
*c* = 20.7565 (8) Å
β = 91.483 (2)°
*V* = 1311.47 (8) Å^3^
*Z* = 4
Mo *K*α radiation
μ = 0.46 mm^−1^
*T* = 296 K
0.24 × 0.18 × 0.15 mm

#### Data collection

Bruker Kappa APEXII CCD diffractometer
Absorption correction: multi-scan (*SADABS*; Bruker, 2005 ▶) *T*_min_ = 0.939, *T*_max_ = 0.940
14693 measured reflections
3269 independent reflections
2513 reflections with *I* > 2σ(*I*)
*R*_int_ = 0.029

#### Refinement

*R*[*F* ^2^ > 2σ(*F* ^2^)] = 0.039
*wR*(*F* ^2^) = 0.108
*S* = 1.02
3269 reflections
182 parameters
H-atom parameters constrained
Δρ_max_ = 0.37 e Å^−3^
Δρ_min_ = −0.36 e Å^−3^

### 

Data collection: *APEX2* (Bruker, 2007 ▶); cell refinement: *SAINT* (Bruker, 2007 ▶); data reduction: *SAINT*; program(s) used to solve structure: *SHELXS97* (Sheldrick, 2008 ▶); program(s) used to refine structure: *SHELXL97* (Sheldrick, 2008 ▶); molecular graphics: *ORTEP-3 for Windows* (Farrugia, 1997 ▶) and *PLATON* (Spek, 2009 ▶); software used to prepare material for publication: *WinGX* (Farrugia, 1999 ▶) and *PLATON*.

## Supplementary Material

Crystal structure: contains datablocks global, I. DOI: 10.1107/S1600536809009787/hb2925sup1.cif

Structure factors: contains datablocks I. DOI: 10.1107/S1600536809009787/hb2925Isup2.hkl

Additional supplementary materials:  crystallographic information; 3D view; checkCIF report

## Figures and Tables

**Table 1:** Hydrogen-bond geometry (Å, °)

*D*—H⋯*A*	*D*—H	H⋯*A*	*D*⋯*A*	*D*—H⋯*A*
O1—H1*O*⋯O2^i^	0.82	1.83	2.648 (2)	178
N1—H1*N*⋯O3^ii^	0.86	2.16	2.898 (2)	144
C4—H4⋯O4	0.93	2.41	3.052 (3)	126
C6—Cl1⋯CgB^iii^	1.73 (1)	3.81 (1)	4.605 (2)	106 (1)
S1—O4⋯CgA^iv^	1.43 (1)	3.14 (1)	4.2532 (9)	134 (1)

Symmetry codes: (i) 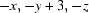; (ii) 
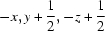
; (iii) 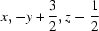; (iv) 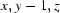. *CgA* and *CgB* are the centroids of the C1–C6 and C8–C13 benzene rings, respectively.

**Fig. 1.**
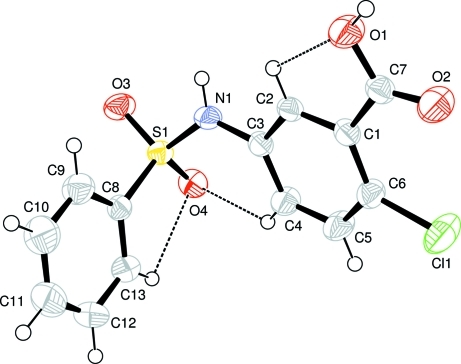
View of (I) with displacement ellipsoids drawn at the 50% probability level. H-atoms are shown by small circles of arbitrary radii. The dotted lines show the intramolecular H-bonds.

**Fig. 2.**
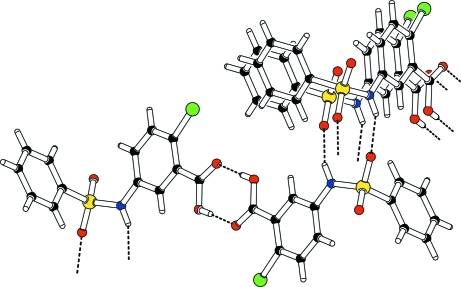
The partial packing diagram of (I) which shows that the molecules are dimerized and linked to each other.

## supplementary crystallographic information

### Comment

Sulfonamide derivatives have been used as antibacterial agents. Recently these type of derivatives (Bouchain *et al.*, 2003) have been reported the as antitumor agents.  As part of our onging studies of sulfonamide (Arshad, Khan *et al.*, 2009) and thiazine related heterocycles (Arshad *et al.*, 2008), we now report the crystal structure of the title compound, (I), (Fig 1).

The crystal structure of 2-chloro-5-(2-iodobenzenesulfonamido)benzoic acid (Arshad, Tahir *et al.*, 2009), (II), has been reported recently. The title compound differs from (II) as there is no iodoine atom on the phenylsulfonyl moiety. Therefore, (II) is the best structure with which the bond distances *etc* can be compared. The title compound consists of dimers due to the carboxylic moiety, forming *R*_2_^2^(8) ring motifs (Bernstein *et al.*, 1995), (Fig 2). These dimers link each other through the N—H···O type of intermolecular H-bonding where the accepter is the SO_2_ moiety (Table 1). The benzene rings A (C1—C6) and B (C8—C13) are oriented at a dihedral angle of 87.07 (6)°. The molecules are stabilized due to intra as well as intermolecular H-bonding and the π-interactions (Table 1).

### Experimental

5-Amino-2-chlorobenzoic acid (1 g, 5.27 mmol) was dissolved in distilled water (10 ml). The pH of the solution was maintained at 8–9 using 1*M*, Na_2_CO_3_ solution. Benzene sulfonyl chloride (0.932 g, 5.27 mmol) was then added to the solution, which was stirred at room temperature until the consumption of all the benzene sulfonyl chloride. During the reaction the pH was again strictly maintained at 8–9 using 1*M*, Na_2_CO_3_. On completion of the reaction the pH was adjusted 1–2, using 1 N HCl under vigorous stirring. The precipitates obtained were filtered off, washed with distilled water and dried. Colourless prisms of (I) were obtained by recrystallization from methanol.

### Refinement

The H-atoms were positioned geometrically, with O-H = 0.82 Å for hydroxy, N—H = 0.86 Å for amine and C—H = 0.93 Å for aromatic H, and constrained to ride on their parent atoms, with U_iso_(H) = 1.2U_eq_(C, N, O).

### Crystal data

**Table:**

C_13_H_10_ClNO_4_S	*F*(000) = 640
*M**_r_* = 311.73	*D*_x_ = 1.579 Mg m^−^^3^
Monoclinic, *P*2_1_/*c*	Mo *K*α radiation, λ = 0.71073 Å
Hall symbol: -P 2ybc	Cell parameters from 2234 reflections
*a* = 11.7139 (4) Å	θ = 2.1–27.0°
*b* = 5.3957 (2) Å	µ = 0.46 mm^−^^1^
*c* = 20.7565 (8) Å	*T* = 296 K
β = 91.483 (2)°	Prismatic, colorless
*V* = 1311.47 (8) Å^3^	0.24 × 0.18 × 0.15 mm
*Z* = 4	

### Data collection

**Table:**

Bruker Kappa APEXII CCD diffractometer	3269 independent reflections
Radiation source: fine-focus sealed tube	2513 reflections with *I* > 2σ(*I*)
graphite	*R*_int_ = 0.029
Detector resolution: 7.40 pixels mm^-1^	θ_max_ = 28.3°, θ_min_ = 2.6°
ω scans	*h* = −15→15
Absorption correction: multi-scan (*SADABS*; Bruker, 2005)	*k* = −7→7
*T*_min_ = 0.939, *T*_max_ = 0.940	*l* = −27→27
14693 measured reflections	

### Refinement

**Table:**

Refinement on *F*^2^	Primary atom site location: structure-invariant direct methods
Least-squares matrix: full	Secondary atom site location: difference Fourier map
*R*[*F*^2^ > 2σ(*F*^2^)] = 0.039	Hydrogen site location: inferred from neighbouring sites
*wR*(*F*^2^) = 0.108	H-atom parameters constrained
*S* = 1.02	*w* = 1/[σ^2^(*F*_o_^2^) + (0.048*P*)^2^ + 0.7097*P*] where *P* = (*F*_o_^2^ + 2*F*_c_^2^)/3
3269 reflections	(Δ/σ)_max_ < 0.001
182 parameters	Δρ_max_ = 0.37 e Å^−^^3^
0 restraints	Δρ_min_ = −0.36 e Å^−^^3^

### Special details

**Table:**

Geometry. Bond distances, angles *etc*. have been calculated using the rounded fractional coordinates. All su's are estimated from the variances of the (full) variance-covariance matrix. The cell e.s.d.'s are taken into account in the estimation of distances, angles and torsion angles
Refinement. Refinement of *F*^2^ against ALL reflections. The weighted *R*-factor *wR* and goodness of fit *S* are based on *F*^2^, conventional *R*-factors *R* are based on *F*, with *F* set to zero for negative *F*^2^. The threshold expression of *F*^2^ > σ(*F*^2^) is used only for calculating *R*-factors(gt) *etc*. and is not relevant to the choice of reflections for refinement. *R*-factors based on *F*^2^ are statistically about twice as large as those based on *F*, and *R*- factors based on ALL data will be even larger.

### Fractional atomic coordinates and isotropic or equivalent isotropic displacement parameters (Å^2^)

**Table:**

	*x*	*y*	*z*	*U*_iso_*/*U*_eq_	
Cl1	0.34738 (5)	1.16021 (13)	−0.01036 (3)	0.0671 (2)	
S1	0.18337 (4)	0.37106 (8)	0.24025 (2)	0.0317 (1)	
O1	−0.00951 (12)	1.2492 (3)	0.05816 (8)	0.0530 (5)	
O2	0.12244 (13)	1.3665 (3)	−0.00925 (8)	0.0557 (5)	
O3	0.08966 (12)	0.2790 (3)	0.27620 (7)	0.0460 (5)	
O4	0.25750 (12)	0.2023 (3)	0.20902 (7)	0.0420 (4)	
N1	0.12278 (13)	0.5510 (3)	0.18667 (8)	0.0370 (5)	
C1	0.16292 (15)	1.0292 (3)	0.06222 (9)	0.0320 (5)	
C2	0.11598 (15)	0.8798 (3)	0.10932 (9)	0.0311 (5)	
C3	0.17784 (15)	0.6911 (3)	0.13923 (9)	0.0313 (5)	
C4	0.28911 (17)	0.6482 (4)	0.12081 (10)	0.0420 (6)	
C5	0.33651 (18)	0.7953 (4)	0.07447 (11)	0.0465 (7)	
C6	0.27543 (17)	0.9845 (4)	0.04525 (10)	0.0385 (6)	
C7	0.09147 (16)	1.2312 (4)	0.03343 (9)	0.0344 (6)	
C8	0.26803 (15)	0.5589 (3)	0.29150 (9)	0.0322 (5)	
C9	0.21566 (19)	0.7353 (4)	0.32856 (11)	0.0484 (7)	
C10	0.2813 (2)	0.8817 (5)	0.36915 (13)	0.0645 (9)	
C11	0.3978 (2)	0.8482 (5)	0.37292 (13)	0.0655 (10)	
C12	0.4499 (2)	0.6723 (5)	0.33606 (12)	0.0558 (8)	
C13	0.38461 (17)	0.5253 (4)	0.29468 (10)	0.0432 (7)	
H1N	0.04957	0.56188	0.18730	0.0444*	
H1O	−0.04283	1.36994	0.04279	0.0636*	
H2	0.04096	0.90730	0.12110	0.0374*	
H4	0.33146	0.52045	0.13970	0.0503*	
H5	0.41138	0.76657	0.06261	0.0558*	
H9	0.13680	0.75516	0.32615	0.0580*	
H10	0.24711	1.00298	0.39397	0.0773*	
H11	0.44182	0.94605	0.40084	0.0786*	
H12	0.52867	0.65190	0.33884	0.0670*	
H13	0.41898	0.40565	0.26941	0.0519*	

### Atomic displacement parameters (Å^2^)

**Table:**

	*U*^11^	*U*^22^	*U*^33^	*U*^12^	*U*^13^	*U*^23^
Cl1	0.0512 (3)	0.0760 (4)	0.0756 (4)	0.0223 (3)	0.0284 (3)	0.0383 (3)
S1	0.0291 (2)	0.0308 (2)	0.0350 (3)	−0.0010 (2)	0.0002 (2)	0.0017 (2)
O1	0.0393 (8)	0.0620 (10)	0.0582 (10)	0.0216 (7)	0.0088 (7)	0.0238 (8)
O2	0.0443 (8)	0.0601 (10)	0.0631 (10)	0.0182 (7)	0.0114 (7)	0.0283 (8)
O3	0.0363 (7)	0.0478 (8)	0.0540 (9)	−0.0098 (6)	0.0038 (6)	0.0115 (7)
O4	0.0442 (8)	0.0354 (7)	0.0462 (8)	0.0065 (6)	−0.0018 (6)	−0.0055 (6)
N1	0.0261 (8)	0.0457 (9)	0.0392 (9)	0.0047 (7)	−0.0004 (6)	0.0085 (7)
C1	0.0325 (9)	0.0349 (9)	0.0284 (9)	0.0056 (7)	−0.0025 (7)	−0.0021 (7)
C2	0.0264 (8)	0.0373 (9)	0.0295 (9)	0.0046 (7)	−0.0020 (7)	−0.0035 (8)
C3	0.0306 (9)	0.0342 (9)	0.0291 (9)	0.0030 (7)	−0.0014 (7)	−0.0019 (7)
C4	0.0353 (10)	0.0448 (11)	0.0460 (12)	0.0138 (9)	0.0047 (8)	0.0102 (9)
C5	0.0342 (10)	0.0539 (13)	0.0519 (13)	0.0159 (9)	0.0114 (9)	0.0118 (10)
C6	0.0356 (10)	0.0430 (11)	0.0372 (10)	0.0060 (8)	0.0059 (8)	0.0050 (9)
C7	0.0322 (9)	0.0382 (10)	0.0326 (10)	0.0053 (8)	−0.0027 (7)	−0.0012 (8)
C8	0.0333 (9)	0.0335 (9)	0.0297 (9)	−0.0038 (7)	0.0016 (7)	0.0022 (7)
C9	0.0438 (12)	0.0503 (12)	0.0512 (13)	0.0002 (10)	0.0056 (10)	−0.0115 (10)
C10	0.0680 (17)	0.0646 (16)	0.0611 (16)	−0.0072 (13)	0.0067 (13)	−0.0284 (13)
C11	0.0665 (17)	0.0720 (18)	0.0577 (15)	−0.0258 (14)	−0.0038 (13)	−0.0170 (13)
C12	0.0362 (11)	0.0722 (16)	0.0587 (14)	−0.0131 (11)	−0.0050 (10)	−0.0017 (12)
C13	0.0351 (10)	0.0513 (12)	0.0433 (12)	−0.0005 (9)	0.0032 (8)	−0.0024 (10)

### Geometric parameters (Å, °)

**Table:**

Cl1—C6	1.730 (2)	C5—C6	1.378 (3)
S1—O3	1.4321 (15)	C8—C13	1.378 (3)
S1—O4	1.4257 (16)	C8—C9	1.378 (3)
S1—N1	1.6255 (17)	C9—C10	1.375 (3)
S1—C8	1.7572 (18)	C10—C11	1.377 (3)
O1—C7	1.305 (2)	C11—C12	1.372 (4)
O2—C7	1.211 (3)	C12—C13	1.385 (3)
O1—H1O	0.8200	C2—H2	0.9300
N1—C3	1.411 (2)	C4—H4	0.9300
N1—H1N	0.8600	C5—H5	0.9300
C1—C6	1.394 (3)	C9—H9	0.9300
C1—C7	1.490 (3)	C10—H10	0.9300
C1—C2	1.391 (3)	C11—H11	0.9300
C2—C3	1.387 (2)	C12—H12	0.9300
C3—C4	1.387 (3)	C13—H13	0.9300
C4—C5	1.375 (3)		
			
O3—S1—O4	119.97 (9)	S1—C8—C9	118.95 (15)
O3—S1—N1	103.69 (9)	S1—C8—C13	119.76 (14)
O3—S1—C8	108.23 (9)	C9—C8—C13	121.29 (18)
O4—S1—N1	109.31 (9)	C8—C9—C10	119.3 (2)
O4—S1—C8	107.63 (9)	C9—C10—C11	119.8 (2)
N1—S1—C8	107.41 (8)	C10—C11—C12	120.9 (2)
C7—O1—H1O	109.00	C11—C12—C13	119.7 (2)
S1—N1—C3	126.74 (13)	C8—C13—C12	119.03 (19)
C3—N1—H1N	117.00	C1—C2—H2	119.00
S1—N1—H1N	117.00	C3—C2—H2	119.00
C2—C1—C6	118.08 (16)	C3—C4—H4	120.00
C2—C1—C7	118.53 (16)	C5—C4—H4	120.00
C6—C1—C7	123.38 (17)	C4—C5—H5	119.00
C1—C2—C3	121.90 (16)	C6—C5—H5	119.00
N1—C3—C2	117.57 (16)	C8—C9—H9	120.00
N1—C3—C4	123.56 (16)	C10—C9—H9	120.00
C2—C3—C4	118.87 (17)	C9—C10—H10	120.00
C3—C4—C5	119.72 (19)	C11—C10—H10	120.00
C4—C5—C6	121.4 (2)	C10—C11—H11	120.00
C1—C6—C5	120.03 (19)	C12—C11—H11	120.00
Cl1—C6—C5	116.31 (16)	C11—C12—H12	120.00
Cl1—C6—C1	123.64 (16)	C13—C12—H12	120.00
O1—C7—O2	122.32 (19)	C8—C13—H13	120.00
O1—C7—C1	113.71 (17)	C12—C13—H13	120.00
O2—C7—C1	123.97 (17)		
			
O3—S1—N1—C3	179.16 (16)	C2—C1—C7—O2	−177.51 (19)
O4—S1—N1—C3	−51.79 (18)	C6—C1—C7—O1	−176.84 (18)
C8—S1—N1—C3	64.71 (17)	C6—C1—C7—O2	3.7 (3)
O3—S1—C8—C9	−47.55 (18)	C1—C2—C3—N1	179.62 (16)
O3—S1—C8—C13	131.51 (16)	C1—C2—C3—C4	−1.0 (3)
O4—S1—C8—C9	−178.57 (16)	N1—C3—C4—C5	−179.45 (19)
O4—S1—C8—C13	0.48 (18)	C2—C3—C4—C5	1.2 (3)
N1—S1—C8—C9	63.83 (18)	C3—C4—C5—C6	−0.6 (3)
N1—S1—C8—C13	−117.12 (16)	C4—C5—C6—Cl1	178.09 (17)
S1—N1—C3—C2	−163.64 (14)	C4—C5—C6—C1	−0.4 (3)
S1—N1—C3—C4	17.0 (3)	S1—C8—C9—C10	179.51 (18)
C6—C1—C2—C3	0.1 (3)	C13—C8—C9—C10	0.5 (3)
C7—C1—C2—C3	−178.76 (17)	S1—C8—C13—C12	−179.00 (17)
C2—C1—C6—Cl1	−177.75 (15)	C9—C8—C13—C12	0.0 (3)
C2—C1—C6—C5	0.6 (3)	C8—C9—C10—C11	−0.9 (4)
C7—C1—C6—Cl1	1.1 (3)	C9—C10—C11—C12	0.8 (4)
C7—C1—C6—C5	179.40 (19)	C10—C11—C12—C13	−0.3 (4)
C2—C1—C7—O1	2.0 (3)	C11—C12—C13—C8	−0.1 (3)

### Hydrogen-bond geometry (Å, °)

**Table:**

*D*—H···*A*	*D*—H	H···*A*	*D*···*A*	*D*—H···*A*
O1—H1O···O2^i^	0.82	1.83	2.648 (2)	178
N1—H1N···O3^ii^	0.86	2.16	2.898 (2)	144
C4—H4···O4	0.93	2.41	3.052 (3)	126
C6—Cl1···CgB^iii^	1.730 (2)	3.8131 (12)	4.605 (2)	106.16 (8)
S1—O4···CgA^iv^	1.4257 (16)	3.1405 (17)	4.2532 (9)	133.77 (8)

Symmetry codes: (i) −*x*, −*y*+3, −*z*; (ii) −*x*, *y*+1/2, −*z*+1/2; (iii) *x*, −*y*+3/2, *z*−1/2; (iv) *x*, *y*−1, *z*.
